# Total Ovarian Volume and Multidimensional Pain in Primary Dysmenorrhea: A Cross-Sectional Ultrasound Study

**DOI:** 10.3390/diagnostics16213635

**Published:** 2026-11-03

**Authors:** Gamze Sönmez Ünal, Nazan Yurtcu

**Affiliations:** 1 Department of Obstetrics and Gynecology, Sivas Numune Hospital, Sivas 58040, Türkiye; 2 Department of Obstetrics and Gynecology, Faculty of Medicine, Sivas Cumhuriyet University, Sivas 58140, Türkiye

**Keywords:** primary dysmenorrhea, ovarian volume, pelvic ultrasonography, Short-Form McGill Pain Questionnaire, pain assessment, receiver-operating characteristic analysis, cross-sectional study

## Abstract

**Background/Objectives:** The relationship between ovarian morphology and primary dysmenorrhea is poorly defined. We examined whether total ovarian volume (TOV) differs by dysmenorrhea status and is associated with multidimensional pain burden. **Methods:** This cross-sectional study included 118 nulliparous women aged 18–24 years, grouped by worst menstrual pain on a 0–10 visual analog scale (VAS ≥ 2, *n* = 76; control, *n* = 42). Pain was assessed with the Short-Form McGill Pain Questionnaire (SF-MPQ), and transabdominal ultrasonography was performed on cycle days 2–5. **Results:** Median TOV was higher with dysmenorrhea (17.04 [12.30–23.48] vs. 11.61 [9.00–15.72] cm^3^; *p* < 0.001). Sensory (17.0 [10.0–21.0] vs. 4.0 [1.0–8.0]), affective (4.0 [2.0–6.0] vs. 0.0 [0.0–2.0]), and total SF-MPQ scores (20.0 [13.2–27.0] vs. 4.5 [1.0–10.2]) were all higher (*p* < 0.001 for each). Each 5 cm^3^ increase in TOV was associated with higher odds of dysmenorrhea after adjustment for clinical covariates (odds ratio 1.764, 95% CI 1.270–2.681; *p* = 0.003), although discrimination was modest (area under the curve 0.718). The association was attenuated when restricted to women with bilateral ovarian volumes ≤ 10 cm^3^. **Conclusions:** Higher TOV accompanied greater multidimensional pain, but the association was not robust to exclusion of larger ovaries and requires external validation.

## 1. Introduction

Primary dysmenorrhea, defined as cyclic menstrual pain without identifiable pelvic pathology, is the most common gynecological complaint among women of reproductive age, with reported frequencies between 45% and 95% depending on the population and diagnostic criteria [1,2,3]. Recurrent menstrual pain can impair physical functioning, school and work productivity, and quality of life [4,5]. Adolescents and young adults carry a particularly high burden, including frequent analgesic use, repeated consultations, and measurable disability [6,7].

Current evidence supports excess endometrial prostaglandin synthesis, principally PGF2α and PGE2, as a central mechanism in primary dysmenorrhea. Increased prostaglandin activity promotes myometrial hypercontractility, uterine ischemia, and peripheral nociceptor sensitization [3,8]. Established clinical risk factors include early menarche, irregular cycles, heavy menstrual flow, low body mass index (BMI), and a positive family history [9]. Whether ovarian morphology, particularly ovarian volume, is associated with dysmenorrhea has received much less attention.

Ovarian volume reflects both stromal tissue and the developing follicular cohort, and ovarian endocrine activity contributes to endometrial prostaglandin regulation [8,10]. Larger ovarian volume could therefore reflect differences in follicular or endocrine activity relevant to menstrual pain, although this mechanism has not been established. Polycystic ovarian morphology has been associated with dysmenorrhea in young women [11], but continuous bilateral ovarian volume measurements in women without a diagnosis of polycystic ovary syndrome have rarely been examined in relation to pain outcomes. Ovarian volume is obtained routinely during pelvic ultrasonography, yet it is rarely considered when young women with menstrual pain are evaluated. To our knowledge, no previous study has examined whether variation in ovarian volume within the range seen in women without polycystic ovary syndrome is related to the presence of dysmenorrhea and to the sensory and affective character of the pain, rather than to its intensity alone.

Distinguishing primary dysmenorrhea from early secondary causes is difficult in young women. Superficial peritoneal endometriosis is usually not visible on ultrasonography, so a normal scan does not exclude the disease [12]. In adolescents and young women with severe dysmenorrhea, careful ultrasonography can nonetheless identify endometriosis or adenomyosis at an early stage [13]. In a prospective ultrasound cohort of 100 symptomatic women with an initially normal examination, 13% had visible endometriosis or adenomyosis lesions at follow-up, whereas most continued to have normal findings [14]. A diagnosis of primary dysmenorrhea made on clinical and sonographic grounds should therefore be regarded as provisional, and some women in this category may have early endometriosis that is not yet detectable.

Pain intensity alone does not capture the full clinical burden of dysmenorrhea. The Short-Form McGill Pain Questionnaire (SF-MPQ) assesses sensory and affective dimensions in addition to pain intensity and has been validated in Turkish [15,16,17]. In the present study, health-related quality of life (HRQoL) and sleep quality were also evaluated as secondary outcomes to characterize the broader symptom burden.

We therefore aimed to (1) compare right, left, and total ovarian volume (ROV, LOV, and TOV) between young nulliparous women with and without dysmenorrhea, defined by a visual analog scale threshold; (2) examine associations between ovarian volume and multidimensional pain, with HRQoL and sleep evaluated as secondary outcomes; and (3) determine whether TOV remained associated with dysmenorrhea after adjustment for the clinical covariates recorded in the study protocol. Exploratory incremental models additionally incorporated HRQoL and sleep measures, and receiver operating characteristic (ROC) analysis was used to describe the discriminative performance of TOV.

## 2. Materials and Methods

### 2.1. Study Design, Setting, and Ethics

This was a single-center cross-sectional study with two comparison groups, reported in accordance with the Strengthening the Reporting of Observational Studies in Epidemiology (STROBE) statement for observational studies [18]. Because exploratory discrimination metrics (AUC, sensitivity, specificity, and predictive values) are also reported, selected Standards for Reporting of Diagnostic Accuracy Studies (STARD) 2015 reporting items were considered when presenting the ROC analysis [19]. Participants were recruited from the Gynecology and Obstetrics Outpatient Clinic of Sivas Numune Hospital between 1 March and 1 May 2026, the two-month window covered by the ethics approval. Questionnaires were administered from the first day of the study. Because ultrasound examinations were restricted to cycle days 2–5 and were performed by one operator, women presenting outside that window were given an appointment for the appropriate cycle phase.

The study was conducted in accordance with the Declaration of Helsinki and approved by the Health Sciences Research Ethics Committee of Sivas Cumhuriyet University (approval no. 26_02_20_77; 8 February 2026). Written informed consent was obtained from all participants before enrollment. The approved protocol covered women aged 18–24 years only; no participant younger than 18 years was enrolled, and every participant provided her own written informed consent.

### 2.2. Participants

Women aged 18–24 years with at least two years of menstrual history were screened consecutively. Inclusion criteria were a menstrual cycle length of 21–35 days verified against a menstrual calendar, nulliparity, never-married status with no history of sexual activity, and no hormonal contraceptive use for at least three months. The age range was selected to focus on adolescents and young adults, in whom primary dysmenorrhea is common and secondary causes of pelvic pain are less prevalent. Nulliparity and the absence of sexual activity were applied as eligibility criteria for two reasons. First, pregnancy, parity and the gynecological conditions associated with sexual activity alter pelvic and ovarian anatomy, so restricting the sample produced a gynecologically homogeneous population in which ovarian volume could be compared without these confounders. Second, transvaginal examination is not undertaken in women without a history of sexual activity in this setting; applying the criterion to the whole sample meant that every participant could be examined by the same transabdominal route, eliminating between-method variability in the primary measurement. Marital status was recorded as a proxy for sexual history rather than as a variable of interest in itself. These criteria restrict the findings to this population and are revisited in the limitations.

Exclusion criteria were clinical or sonographic signs of endometriosis or adenomyosis; polycystic ovary syndrome; uterine leiomyoma; pelvic inflammatory disease; irritable bowel syndrome; chronic or recurrent cystitis or bladder pain; any other identifiable pelvic pathology capable of causing secondary dysmenorrhea; previous pelvic or abdominal surgery; chronic systemic disease; current pregnancy; and lactation. Mean cycle length had to remain within 21–35 days for eligibility. Cycle-to-cycle regularity within that range was recorded separately and treated as a covariate.

Eligibility was assessed by combining a structured medical, gynecological, gastrointestinal, and urinary history; general physical and abdominal examination; transabdominal pelvic ultrasonography (Section 2.4); serum androgen measurement; and urinalysis. Because no participant had a history of sexual activity, speculum and bimanual pelvic examinations were not performed. Endometriosis and adenomyosis were considered absent when there were no clinical signs and no ovarian endometrioma or sonographic feature of adenomyosis [13]; superficial peritoneal disease cannot be excluded by this approach [12] (Section Strengths and Limitations). Polycystic ovary syndrome (PCOS) was assessed according to the 2023 international evidence-based guideline [20], using clinical, biochemical, and sonographic criteria. Because a cycle length of 21–35 days was an inclusion criterion, women with ovulatory dysfunction as defined in that guideline were not enrolled. Clinical hyperandrogenism was evaluated with the modified Ferriman–Gallwey score and by examination for acne and androgenic alopecia [20]. Biochemical hyperandrogenism was assessed by measuring serum total testosterone and dehydroepiandrosterone sulfate in all participants, interpreted against the institutional laboratory reference ranges. Women who met the guideline criteria for PCOS were excluded. Pelvic inflammatory disease was considered unlikely in the absence of sexual activity and was further excluded by clinical history, the absence of abdominal tenderness, and the absence of hydrosalpinx or adnexal fluid collections on ultrasonography. Irritable bowel syndrome was screened during the interview with the Rome IV diagnostic criteria, as recommended in current clinical guidance [21]. Chronic or recurrent cystitis and bladder pain were screened by urinary symptom history (dysuria, frequency, urgency, and suprapubic pain related to bladder filling), and urinalysis was performed in all participants; women with urinary symptoms or abnormal urinalysis findings were excluded.

The study protocol defined the dysmenorrhea group clinically, as recurrent menstrual pain of at least six months’ duration in the absence of pelvic pathology. For analysis this was operationalized on the visual analog scale (VAS): women reporting worst menstrual pain of ≥2 on the 0–10 VAS were classified in the dysmenorrhea group and those with VAS < 2 as controls. The numerical threshold was set during analysis and was not specified in the protocol. A dichotomous self-report item was also recorded; the two classifications disagreed for 23 participants, and the self-report classification was retained for a sensitivity analysis. Because VAS defined group membership, it was not analyzed as an independent between-group outcome. Multidimensional pain was evaluated separately using the SF-MPQ.

Recruitment followed a two-visit procedure. Women attending the outpatient clinic were approached consecutively and, after consent, completed the full questionnaire set at that first visit: the VAS, the SF-MPQ, the 12-Item Short Form Health Survey (SF-12), and the Pittsburgh Sleep Quality Index (PSQI), together with demographic and menstrual history. Ovarian volume varies across the cycle, so imaging was not performed at that visit. Women who happened to present during the early follicular phase were scanned that same week; the rest were given an appointment and recalled for scanning on cycle days 2–5 of their next menstrual period. Therefore, the interval between the questionnaire and imaging ranged from a few days to approximately one cycle. Questionnaires were self-completed by the participant in a private examination room without a parent, guardian or companion present, and were returned directly to the investigator. Forms carried a study code rather than a name, and the code-to-identity key was held separately from the analysis dataset by the corresponding author.

**Figure 1:**
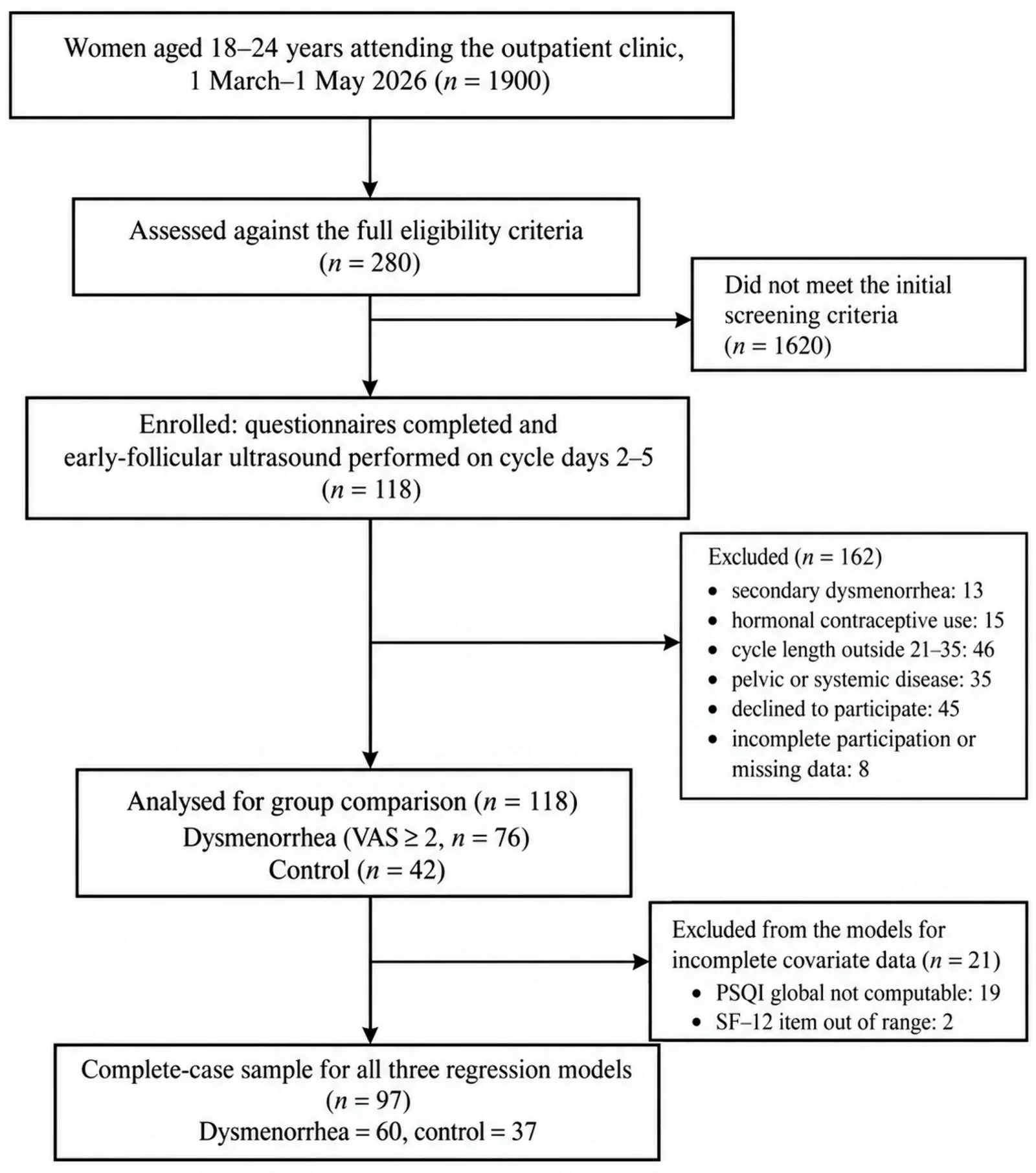
Participant flow diagram. Screening counts are taken from the recruitment log; all counts from enrollment onwards are generated by the analysis script. Group assignment was made on the visual analog scale (VAS ≥ 2). Twenty-one participants were excluded from the regression models for incomplete covariate data, 19 of them because a PSQI global score could not be computed.

Approximately 1900 women aged 18–24 years attended the clinic during the two-month recruitment period. Of these, 280 met the initial screening criteria and were assessed against the full eligibility criteria; 162 were excluded for a menstrual cycle length outside the 21–35 day range (46), declining to participate (45), pelvic or systemic disease (35), hormonal contraceptive use (15), secondary dysmenorrhea (13), and incomplete participation or missing data (8). This left 118 participants who completed both the questionnaire set and the ultrasound examination (76 classified as having dysmenorrhea, 42 as controls). A single age entry was corrected against the source form during data checking; no record was excluded on eligibility grounds after enrollment. The participant flow is shown in Figure 1. The screening counts were obtained from the recruitment log; every count from enrollment onwards is generated by the analysis script.

Sample size. The available protocol documentation specified a minimum sample size of 35 participants per group (70 in total), calculated using PASS 2008 (NCSS, Kaysville, UT, USA) with a two-sided α of 0.05 and statistical power of 0.90. The protocol did not record the underlying expected between-group mean difference or common standard deviation. For an equal-allocation two-sample comparison of means, the recorded sample size, α, and power imply a standardized effect size of approximately Cohen’s d = 0.79. This value is therefore a retrospective reconstruction of the original calculation rather than a prespecified effect-size assumption. Recruitment continued throughout the prespecified enrollment period, and 118 participants were ultimately enrolled. The multivariable regression analyses were exploratory and were not part of the original sample-size calculation.

### 2.3. Outcome Measures

Pain intensity was rated on a 0–10 VAS for the most painful day of the current or immediately preceding cycle. Participants were asked to rate the pain as experienced before any analgesic had been taken. Analgesic use was recorded separately with a single item asking whether the participant ever took pain medication during menstruation; the item did not specify the indication or a particular cycle. In 3 participants the scale had been entered in millimeters, and those values were rescaled; this was the only alteration made to the pain data.

Multidimensional pain was assessed using the SF-MPQ [15,16], validated in Turkish [17]. Each of the 15 descriptors is rated 0–3, giving a total of 0–45 covering sensory (items 1–11) and affective (items 12–15) dimensions. A total of 2 item responses fell outside the 0–3 range and were treated as missing rather than truncated to the scale maximum, leaving 2 participants without a complete SF-MPQ total.

HRQoL was assessed using the SF-12 [22] and the Turkish version validated by Soylu and Kütük [23]. Each SF-12v1 item was linearly transformed to a 0–100 scale oriented toward better health, and study-specific physical and mental summary indices were calculated as the unweighted means of their six constituent items. The proprietary norm-based PCS-12/MCS-12 algorithm was not applied; therefore, these study-specific indices should not be interpreted as conventional norm-based PCS-12 or MCS-12 scores. Four item responses fell outside the SF-12v1 response range and were set to missing before scoring. Higher values indicate better health.

Sleep quality was assessed using the PSQI [24,25], scored according to the original Buysse algorithm: seven components are rated 0–3 and summed to a global score of 0–21, with values above 5 indicating poor sleep. One sleep-duration entry had been recorded in hh:mm rather than decimal format and was converted. A global PSQI score requires all seven components; 19 participants lacked usable bed-time and wake-time data for habitual sleep efficiency (component 4) and therefore had no global score. PSQI analyses were based on 99 participants and were considered secondary and exploratory.

### 2.4. Ovarian Volume Measurement

**Figure 2:**
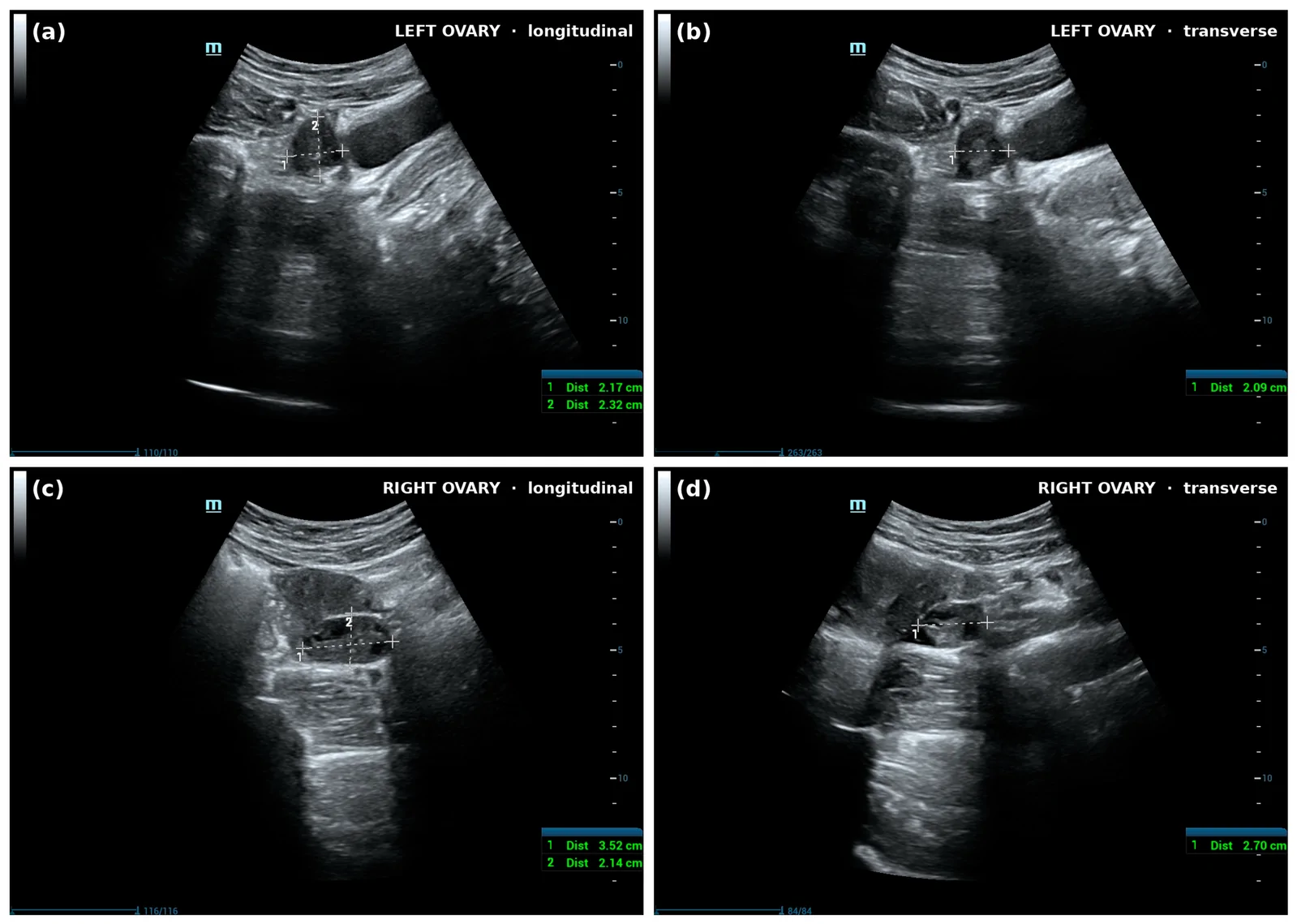
Transabdominal ultrasonographic measurement of ovarian volume in a participant without dysmenorrhea. (**a**) Left ovary, longitudinal plane, with the long-axis (1, 2.17 cm) and anteroposterior (2, 2.32 cm) diameters marked by electronic calipers; (**b**) left ovary, transverse plane obtained after a 90° probe rotation, showing the width (2.09 cm); left ovarian volume 5.50 cm^3^. (**c**) Right ovary, longitudinal plane (1, 3.52 cm; 2, 2.14 cm) and (**d**) transverse plane (2.70 cm); right ovarian volume 10.64 cm^3^. Total ovarian volume 16.14 cm^3^. Volumes were calculated as V = 0.523 × length × width × height. Images were acquired with a full bladder on a Mindray Resona R9 system; the depth scale is shown on the right of each panel.

All pelvic ultrasound examinations were performed by one experienced operator (G.S.Ü.) on a Mindray Resona R9 system with an SC6-1U convex probe (Mindray, Shenzhen, China), by the transabdominal route with a full bladder. Every participant was nulliparous, never married, and reported no history of sexual activity, so the transvaginal route was not offered to anyone and transabdominal scanning was the only approach used. Transabdominal ultrasonography has been shown to be feasible for assessing ovarian morphology in young women [10]. Confining the study to one approach removes between-method variability, but at the cost of some measurement precision, which is taken up in the limitations. Ovarian volume was calculated with the prolate ellipsoid formula, V (cm^3^) = 0.523 × length × width × height [26]. For orientation, ovarian volume in healthy women of reproductive age is usually reported in the region of 4–10 cm^3^, varying with age and body size [27]. ROV and LOV were measured separately and TOV defined as their sum. All examinations were performed on cycle days 2–5, the appointment for which had been set at the recruitment visit, so that follicular recruitment had not yet advanced and cycle-phase variation in ovarian volume was minimized. Figure 2 was acquired on the same ultrasound system and with the same full-bladder transabdominal protocol used throughout the study, and demonstrates caliper placement and the application of the prolate ellipsoid formula. The images were exported with all patient-identifying header information removed, and the archived examination carries no name, hospital record number, accession number or examination date in the published panels; written consent for publication of the de-identified images was obtained from the participant concerned. The operator was aware of symptom status; this is addressed in the limitations.

### 2.5. Statistical Analysis

All analyses were performed from the locked raw dataset using a reproducible script, and the reported results were generated directly from this analysis.

Distributional assumptions were checked with the Shapiro–Wilk test together with histograms and Q–Q plots. Continuous variables that departed from normality in either group were compared with the Mann–Whitney U test and are summarized as median (interquartile range), with the rank-biserial correlation as the effect size; normally distributed variables were compared with the independent-samples t test and summarized as mean ± SD. Categorical variables were compared using the chi-square test or Fisher’s exact test when any expected cell count fell below five. The association between ovarian volume and each outcome was quantified using Spearman’s ρ.

Logistic regression with odds ratios and 95% confidence intervals was specified in the protocol. Three nested models were constructed for this analysis, with dysmenorrhea status as the outcome. Model A was the primary adjusted model and included TOV per 5 cm^3^ increment, age, BMI, age at menarche, and cycle regularity. Models B and C were exploratory incremental models: Model B added the study-specific SF-12 physical and mental summary indices, and Model C additionally added the PSQI global score. All three models were fitted on the same complete-case sample (*n* = 97; 21 participants excluded for incomplete covariate data), allowing direct comparison of Akaike information criterion (AIC) values and likelihood-ratio tests. Model fit is reported using AIC, McFadden pseudo-R^2^, and Nagelkerke pseudo-R^2^; nested models were compared using likelihood-ratio tests. Multicollinearity was assessed using variance inflation factors. Because HRQoL and sleep may be consequences or correlates of dysmenorrhea rather than confounders, Models B and C were not treated as primary causal adjustment models.

Internal validity of each model was assessed using bootstrap optimism correction over 1000 resamples: each bootstrap model was applied both to its own resample and to the original data, and the mean difference in AUC was subtracted from the apparent AUC [28,29]. ROC analysis was not part of the protocol and is reported as exploratory. It used 2000 bootstrap resamples for the confidence interval, and the Youden index identified the optimal cut-off. Predictive values depend on the sample prevalence of 64.4% and should not be generalized to populations with different prevalence. PSQI component-level comparisons were exploratory; no adjustment for multiple comparisons was applied, and no primary inference was based on individual component *p* values. Significance was set at α = 0.05, two-tailed. Analyses were performed in R v4.3.2 (R Foundation for Statistical Computing, Vienna, Austria) with the pROC, car, and boot packages.

## 3. Results

### 3.1. Participant Characteristics

In total, 118 women were analyzed: 76 classified as having primary dysmenorrhea and 42 as controls, all nulliparous, never married, sexually inactive, and aged 18–24 years. Baseline characteristics and outcome measures are shown in Table 1. The dysmenorrhea group was slightly older (*p* = 0.007); the groups did not differ in BMI (*p* = 0.198), age at menarche (*p* = 0.815), cycle regularity (*p* = 0.662), or family history of dysmenorrhea (*p* = 0.240). Analgesic use during menstruation was substantially more common in the dysmenorrhea group (59 (77.6%) vs. 13 (31.0%); *p* < 0.001).

### 3.2. Ovarian Volumes

**Figure 3:**
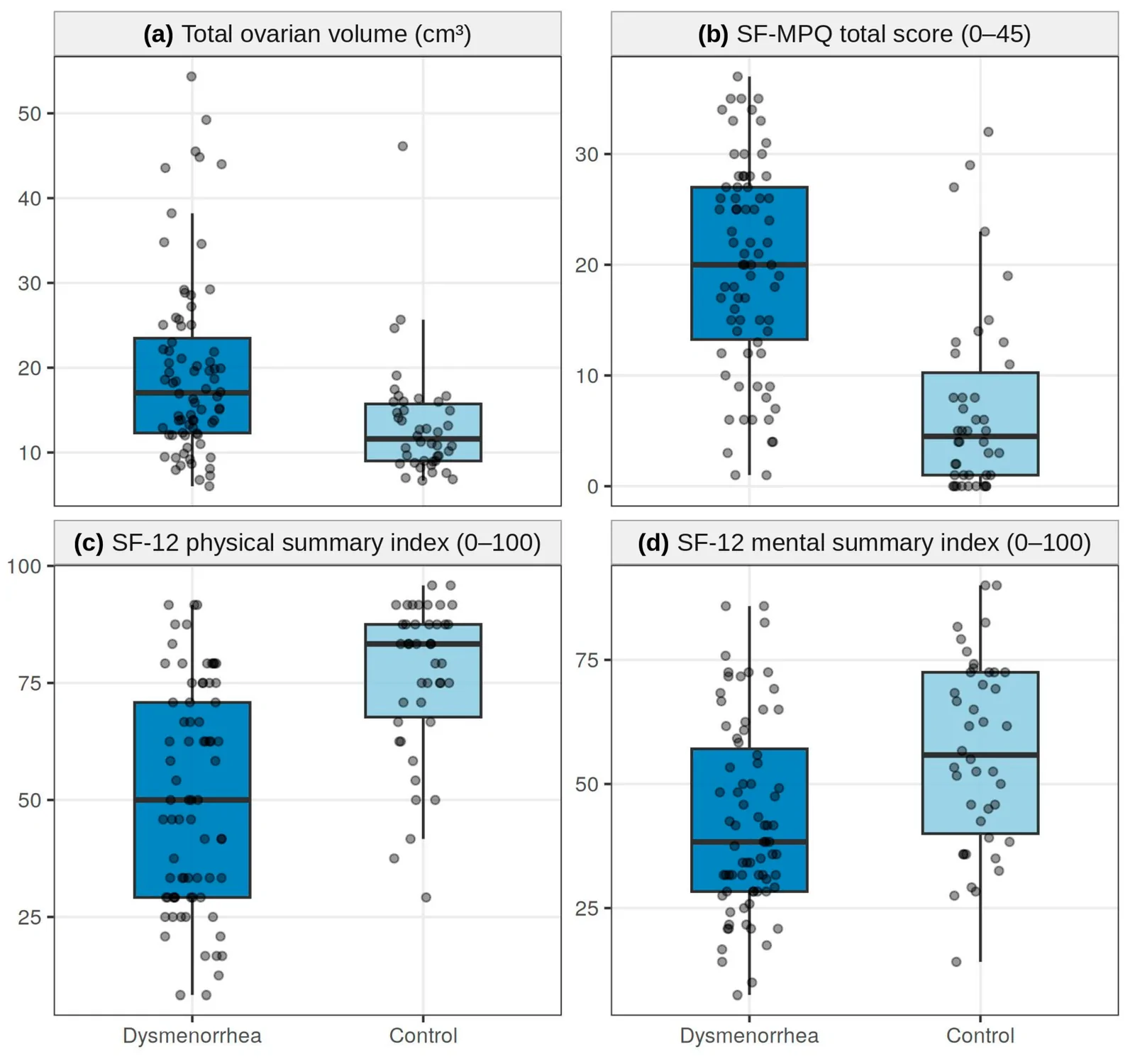
Group comparisons for (**a**) total ovarian volume, (**b**) SF-MPQ total score, and the secondary HRQoL measures (**c**) SF-12 physical summary index and (**d**) SF-12 mental summary index. Box plots show medians and interquartile ranges with jittered individual observations. Between-group tests and effect sizes are given in Table 1 and Table 2.

All three volume parameters were larger in the dysmenorrhea group (Table 2, Figure 3a). The median difference in TOV was 5.43 cm^3^ (17.04 (12.30–23.48) vs. 11.61 (9.00–15.72) cm^3^; *p* < 0.001; r = 0.437). ROV and LOV showed the same direction and comparable effect sizes (r = 0.377 and 0.397 respectively).

### 3.3. Associations Between Ovarian Volume and Clinical Outcomes

**Figure 4:**
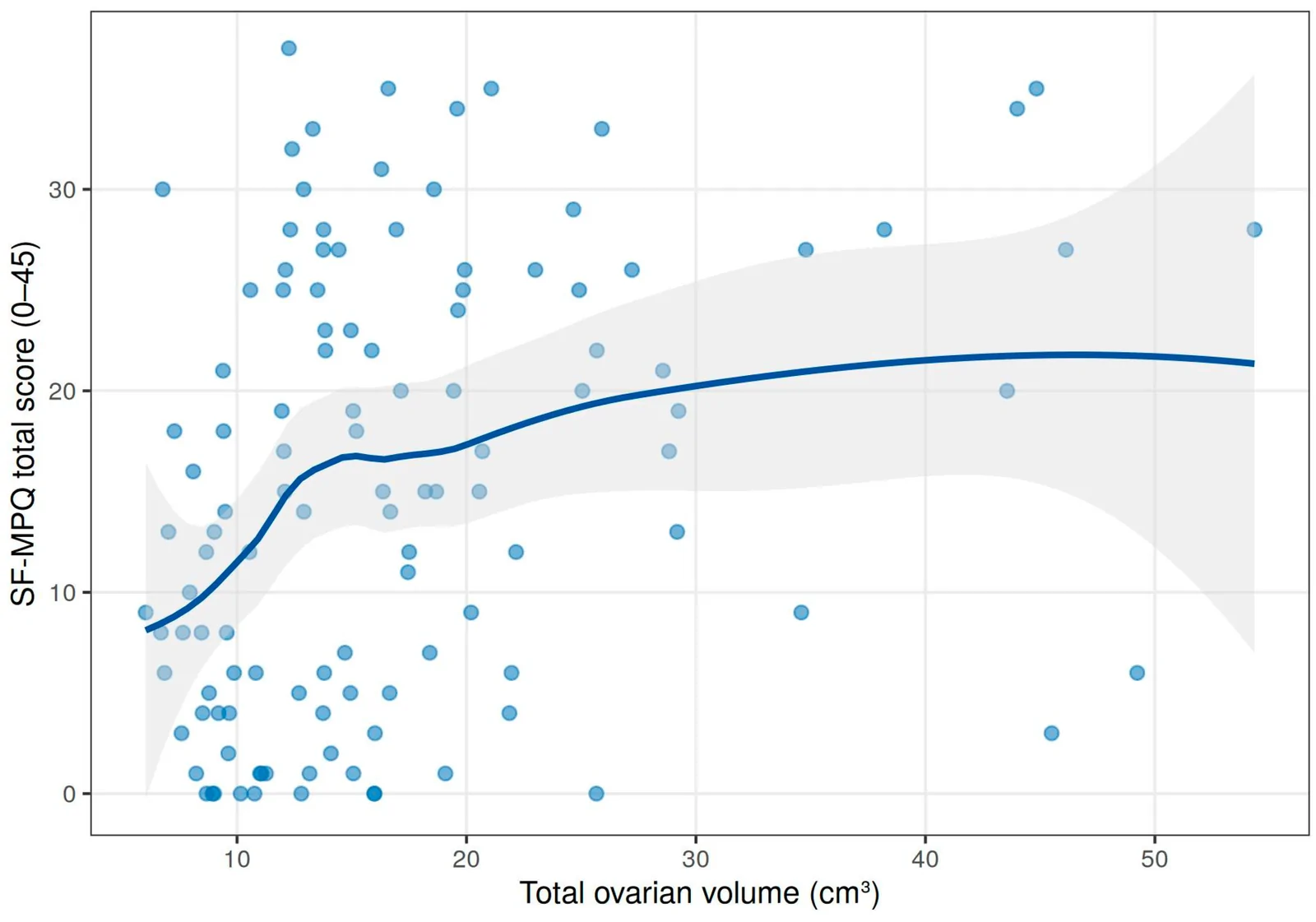
Total ovarian volume against SF-MPQ total score, with a locally weighted (LOESS) trend line. The SF-MPQ is used here rather than the VAS because the VAS defines group membership. Spearman ρ = 0.354, *p* < 0.001. Blue circles represent individual participants, the solid blue line indicates the fitted smoothed trend, and the shaded area represents the uncertainty band around the fitted curve.

Spearman correlations are given in Table 3. TOV correlated positively with SF-MPQ total (ρ = 0.354, *p* < 0.001; Figure 4) and with VAS (ρ = 0.334, *p* < 0.001), and negatively with the study-specific SF-12 physical summary index (ρ = −0.318, *p* < 0.001). Correlations with the mental summary index (ρ = −0.163, *p* = 0.079) and PSQI global score (ρ = 0.203, *p* = 0.044, *n* = 99) were weaker.

### 3.4. Multivariable Logistic Regression

Model A was the primary adjusted model and was fitted on the same complete-case sample used for all nested models (*n* = 97; 60 with dysmenorrhea and 37 controls) (Table 4). Each 5 cm^3^ increment in TOV was associated with higher odds of dysmenorrhea (odds ratio [OR] 1.764, 95% confidence interval [CI] 1.270–2.681; *p* = 0.003). Age was also significantly associated with dysmenorrhea (OR 1.325, 95% CI 1.082–1.654; *p* = 0.009). BMI, age at menarche, and cycle regularity were not significant. In Model B, adding the SF-12 indices improved model fit (AIC 120.1 to 102.9; likelihood-ratio test *p* < 0.001); TOV (OR 1.559, 95% CI 1.153–2.303; *p* = 0.010), age (*p* = 0.022), and the physical summary index (OR 0.951, 95% CI 0.917–0.980; *p* = 0.003) remained associated with dysmenorrhea. Adding PSQI in Model C did not significantly improve fit over Model B (likelihood-ratio test *p* = 0.085). In Model C, TOV (OR 1.626, 95% CI 1.190–2.464; *p* = 0.007), age (*p* = 0.025), and the physical summary index (OR 0.948, 95% CI 0.911–0.978; *p* = 0.002) remained significant, whereas PSQI did not (*p* = 0.092). Maximum variance inflation factor across models was 1.45.

Three post hoc sensitivity analyses were applied to Model C. Using the self-reported group definition instead of the VAS rule produced a similar TOV estimate (OR 1.498, 95% CI 1.078–2.348; *p* = 0.035). Excluding the one participant whose two ovarian volumes differed by more than fivefold also had little effect (*n* = 96; OR 1.605, 95% CI 1.164–2.445; *p* = 0.010). In the analysis restricted to women with bilateral ovarian volumes ≤ 10 cm^3^, the sample decreased to 65 and the TOV estimate became imprecise and was no longer statistically significant (OR 1.613, 95% CI 0.504–5.536; *p* = 0.426). This sensitivity analysis therefore does not establish that the association is independent of larger ovarian morphology.

### 3.5. Diagnostic Performance of Total Ovarian Volume

**Figure 5:**
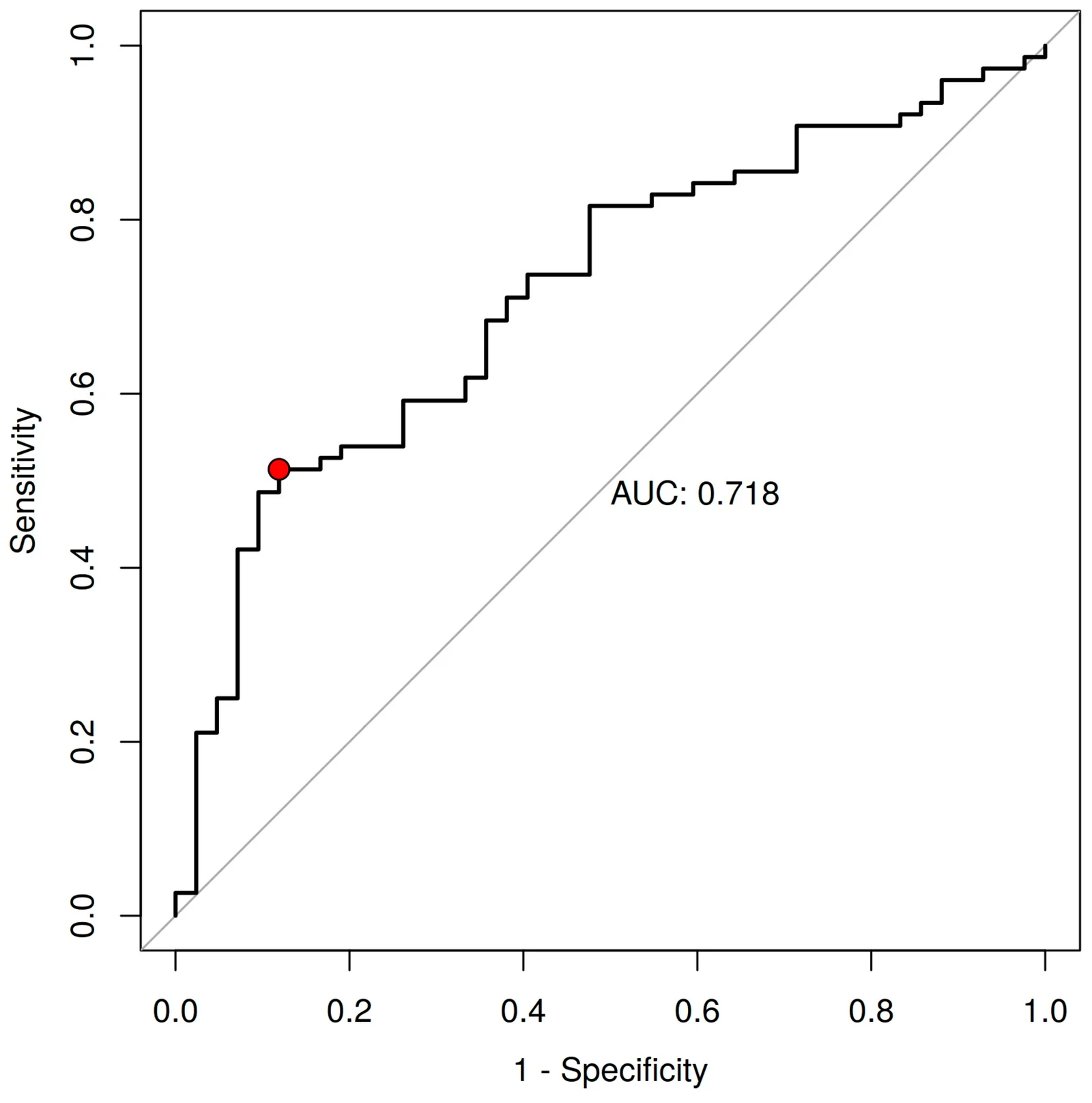
ROC curve for total ovarian volume as a marker of primary dysmenorrhea. AUC = 0.718 (0.621–0.814). The solid black line shows the empirical ROC curve, and the grey diagonal line indicates chance-level discrimination (AUC = 0.5). The red circle marks the Youden-optimal cut-off (16.81 cm^3^; sensitivity 51.3%, specificity 88.1%).

Considered alone, TOV discriminated between groups with an AUC of 0.718 (95% CI 0.621–0.814) (Figure 5). The Youden-optimal cut-off was 16.81 cm^3^, giving sensitivity 51.3% and specificity 88.1%. Positive and negative predictive values (88.6% and 50.0%) reflect the sample prevalence of 64.4% and would differ in an unselected clinic population. The optimism-corrected AUC of Model C was 0.828 (apparent AUC 0.876; optimism 0.048).

### 3.6. Multidimensional Pain

SF-MPQ total scores were substantially higher in the dysmenorrhea group (20.0 [13.2–27.0] vs. 4.5 [1.0–10.2]; *p* < 0.001; r = 0.696; Figure 3b). Both SF-MPQ dimensions differed between groups: the sensory subscore (items 1–11) was 17.0 [10.0–21.0] vs. 4.0 [1.0–8.0], and the affective subscore (items 12–15) was 4.0 [2.0–6.0] vs. 0.0 [0.0–2.0] (*p* < 0.001 for both; rank-biserial r = 0.727 and 0.510, respectively). VAS and SF-MPQ were moderately to strongly correlated (ρ = 0.636; *p* < 0.001), supporting convergence between the two pain measures while indicating that they capture partially distinct aspects of pain.

### 3.7. Health-Related Quality of Life

As a secondary analysis, both study-specific SF-12 indices were lower in the dysmenorrhea group (Figure 3c,d). The difference was larger for the physical index (50.0 [29.2–70.8] vs. 83.3 [67.7–87.5]; *p* < 0.001; r = 0.620) than for the mental index (38.3 [28.3–57.1] vs. 55.8 [40.0–72.5]; *p* < 0.001; r = 0.410). In the incremental models, the physical index remained associated with dysmenorrhea, whereas the mental index did not. These findings should be interpreted as secondary because the study-specific indices are not conventional norm-based PCS-12/MCS-12 scores and may reflect consequences of dysmenorrhea rather than confounding factors.

### 3.8. Sleep Quality

Global PSQI scores did not differ between groups (7.0 [5.0–9.0] vs. 6.0 [5.0–9.0]; *p* = 0.449; r = 0.091; Figure S1), and poor sleep quality (PSQI > 5) was common in both groups (41 [66.1%] vs. 20 [54.1%]; OR 1.65, 95% CI 0.66–4.14; *p* = 0.287). In exploratory component-level analyses, daytime dysfunction showed the largest between-group difference (*p* = 0.001; r = 0.345), whereas the remaining components showed small or no differences (Table 5, Figure S2). These component-level findings were not used for primary inference.

## 4. Discussion

This study identified a consistent association between ovarian volume and dysmenorrhea in young nulliparous women. ROV, LOV, and TOV were larger in the dysmenorrhea group, and TOV correlated with multidimensional pain measured by the SF-MPQ. In the primary adjusted model, TOV remained associated with dysmenorrhea after adjustment for age, BMI, age at menarche, and cycle regularity. TOV alone, however, showed only modest discriminative performance. HRQoL and sleep findings were secondary: physical and mental SF-12-derived indices were lower in the dysmenorrhea group, whereas global PSQI did not differ.

A possible biological explanation involves the estrogen–prostaglandin axis. Increased endometrial prostaglandin activity is central to the pathophysiology of primary dysmenorrhea [3,8], and ovarian volume reflects stromal tissue and follicular content [10]. Larger ovarian volume could therefore reflect differences in follicular or endocrine activity relevant to menstrual pain. However, estradiol, progesterone, prostaglandin metabolites, and ovulatory status were not measured, so this mechanism remains hypothetical. The previously reported association between polycystic ovarian morphology and dysmenorrhea in young women [11] provides relevant context but does not establish a causal pathway.

SF-MPQ scores indicated that the between-group difference extended beyond pain intensity to sensory and affective pain dimensions, with a large effect size. The moderate-to-strong correlation between VAS and SF-MPQ supports overlap between the instruments while also suggesting that they are not interchangeable. Because VAS was used to define group membership, the SF-MPQ provides the more informative independent pain comparison in this dataset.

Primary dysmenorrhea lies within the broader differential diagnosis of pelvic pain in young women. Endometriosis, adenomyosis, irritable bowel syndrome, bladder pain syndrome/interstitial cystitis, and myofascial pelvic floor pain can produce cyclic or acyclic pelvic pain, frequently coexist, and may share centrally mediated pain mechanisms [30]. These conditions were screened for by history, abdominal examination, transabdominal ultrasonography, the Rome IV criteria, and urinalysis, but not all of them could be excluded with certainty. Superficial peritoneal endometriosis is not detectable by ultrasonography [12], transabdominal imaging is less sensitive than transvaginal imaging for small lesions and early adenomyosis, and pelvic floor muscle function was not examined. Unrecognized secondary pathology or a coexisting visceral pain condition in some women of the dysmenorrhea group could have raised pain scores independently of ovarian volume, and this possibility should be kept in mind when the association is interpreted. Some women in the dysmenorrhea group may also have had early endometriosis that was not yet detectable; in the prospective cohort cited in the Introduction, a minority of symptomatic women with an initially normal scan later developed visible lesions [14]. If such women were included, they could have contributed both to the higher pain scores and to the observed association with ovarian volume. Long-term follow-up with repeat ultrasonography of women classified as having primary dysmenorrhea would help to clarify this question.

The HRQoL findings should be interpreted as secondary. Both study-specific SF-12 indices were lower in the dysmenorrhea group, and the physical index remained associated with dysmenorrhea in the incremental models. However, these indices were derived using a study-specific 0–100 scoring approach rather than the conventional norm-based PCS-12/MCS-12 algorithm, and HRQoL may itself be a consequence of recurrent menstrual pain. Reduced menstruation-related quality of life in young women with primary dysmenorrhea has also been reported previously [31]. The lack of a group difference in global PSQI suggests that habitual monthly sleep quality was not clearly associated with dysmenorrhea in this sample. The component-level PSQI findings, including daytime dysfunction, are exploratory and require confirmation with cycle-anchored sleep measures. Previous studies in young women have also reported associations between sleep characteristics and dysmenorrhea [32,33].

Thirteen of the 42 controls (31.0%) reported analgesic use during menstruation despite VAS scores of 0–1. Because the VAS was rated for pain before any analgesic had been taken, this finding is unlikely to reflect pain masked by medication. Two features of the data collection may explain it. First, the analgesic item asked whether the participant ever took pain medication during menstruation and did not specify the indication, so use for other perimenstrual complaints, such as headache or low back pain, would also have been recorded. Second, the item referred to habitual use, whereas the VAS referred to the most painful day of the current or preceding cycle; occasional use during an earlier, more painful cycle would therefore be captured by the analgesic item but not by the VAS. Self-medication with over-the-counter analgesics is common among young women [1]. Analgesic use was not used to define the groups and was not included as a covariate.

TOV alone yielded an AUC of 0.718, with relatively high specificity but limited sensitivity at the data-derived Youden cut-off. This level of discrimination is insufficient for TOV to be considered a standalone diagnostic test. Primary dysmenorrhea remains a clinical diagnosis based on history and exclusion of secondary pelvic pathology. The cut-off reported here is sample-specific and should not be used as a clinical threshold.

The bootstrap-corrected performance of the multivariable models was higher than that of TOV alone, but the incremental models incorporated HRQoL and sleep measures that may reflect the consequences of dysmenorrhea. Model A is the primary adjusted model for interpreting the association between TOV and dysmenorrhea; Models B and C should therefore be interpreted as exploratory analyses.

### Strengths and Limitations

The main strengths are the uniform ultrasound protocol—one operator, one machine, one probe, one route, and a fixed cycle-day window—the use of effect sizes and confidence intervals, a single primary adjusted model fitted on protocol-recorded covariates, and bootstrap internal validation. Fitting the nested models on the same complete-case sample also allowed valid comparison of AIC values and likelihood-ratio tests.

Several limitations constrain interpretation. First, the cross-sectional design precludes causal inference and temporal ordering. Second, 41 of 118 participants had at least one ovary > 10 cm^3^, and antral follicle counts were not recorded. When the analysis was restricted to women with bilateral ovarian volumes ≤ 10 cm^3^, the TOV estimate became imprecise and was no longer statistically significant; therefore, the primary association cannot be considered independent of larger ovarian morphology. Third, group status was defined by a VAS threshold that was set during analysis rather than in the protocol, which both prevents VAS from serving as an independent outcome and leaves the cut-off open to revision; the self-report classification is therefore reported as a sensitivity analysis. Fourth, the SF-12-derived indices used a study-specific 0–100 scoring method and are not equivalent to conventional norm-based PCS-12/MCS-12 scores. Fifth, the incremental models included several predictors in only 97 complete cases and may be unstable despite bootstrap validation. The ultrasound operator was not blinded to symptom status, intraobserver reliability was not quantified, and transabdominal imaging is less precise than transvaginal imaging for ovarian measurement. Because static images were not archived at the time of the index examinations, the recorded diameters could not be re-measured retrospectively or reviewed by a second observer. Ovarian volumes were derived from two-dimensional measurements with the prolate ellipsoid formula, as the transducer used throughout the study did not support three-dimensional acquisition; two- and three-dimensional methods are not interchangeable [34]. Apart from serum androgens, hormonal assays and ovulation measures were not available. Although polycystic ovary syndrome was excluded on clinical, biochemical, and sonographic grounds, polycystic ovarian morphology without the full syndrome could not be excluded, because antral follicle counts were not recorded; this is one reason the analysis restricted to women with bilateral ovarian volumes ≤ 10 cm^3^ is reported. Superficial peritoneal endometriosis cannot be excluded without laparoscopy, pelvic floor muscle function was not examined, and the analgesic item did not record the indication for use. Complete-case modeling excluded 21 participants, and questionnaires and imaging were obtained at separate visits. Recruitment ran over two months in spring; because PSQI scores vary seasonally, an imbalance in the calendar distribution of the two groups could introduce differential measurement error in the sleep outcome, although a window this short makes a large effect unlikely. Finally, the single-center sample was restricted to a narrow age range, limiting generalizability.

## 5. Conclusions

In young nulliparous women, higher total ovarian volume was associated with dysmenorrhea and a greater multidimensional pain burden. TOV remained associated with dysmenorrhea in the primary model after adjustment for age, BMI, age at menarche, and cycle regularity, but its standalone discriminative performance was modest. HRQoL differences were secondary findings, and global sleep quality did not differ between groups. The association was attenuated and no longer statistically significant when the analysis was restricted to women with bilateral ovarian volumes ≤ 10 cm^3^. These findings are hypothesis-generating and require prospective, blinded external validation before clinical application.

## Figures and Tables

**Table 1:** Baseline characteristics and clinical outcome measures by dysmenorrhea status.

Variable	Dysmenorrhea (76)	Control (42)	*p* Value	r
Age (years)	22.0 (20.0–23.0)	21.0 (18.2–22.0)	0.007	0.295
BMI (kg/m^2^)	22.8 (20.8–25.1)	21.8 (19.6–24.6)	0.198	0.144
Age at menarche (years)	13.0 (12.0–14.0)	13.0 (12.0–14.0)	0.815	0.025
Regular menstrual cycle, *n* (%)	57 (75.0%)	33 (78.6%)	0.662	—
Family history of dysmenorrhea, *n* (%)	50 (65.8%)	32 (76.2%)	0.240	—
Analgesic use during menstruation, *n* (%)	59 (77.6%)	13 (31.0%)	<0.001	—
VAS score (0–10), group-defining	7.0 (4.0–8.0)	0.0 (0.0–1.0)	—	—
SF-MPQ total (0–45)	20.0 (13.2–27.0)	4.5 (1.0–10.2)	<0.001	0.696
SF-12 physical summary index (study-specific)	50.0 (29.2–70.8)	83.3 (67.7–87.5)	<0.001	0.620
SF-12 mental summary index (study-specific)	38.3 (28.3–57.1)	55.8 (40.0–72.5)	<0.001	0.410
PSQI global score (0–21)	7.0 (5.0–9.0)	6.0 (5.0–9.0)	0.449	0.091

The VAS defines group membership and is shown for description only; no between-group test is reported for it. Continuous data are median (IQR) unless stated otherwise. BMI = body mass index; IQR = interquartile range; PSQI = Pittsburgh Sleep Quality Index; SF-12 = 12-Item Short Form Health Survey; SF-MPQ = Short-Form McGill Pain Questionnaire; VAS = visual analog scale. The SF-12 physical and mental summary indices are study-specific 0–100 scores and are not conventional norm-based PCS-12/MCS-12 scores; higher values indicate better health. PSQI global scores are based on 99 participants with all seven components available. *p* Values are from the Mann–Whitney U test for continuous variables and the chi-square or Fisher exact test for categorical variables; r is the rank-biserial correlation. Analgesic use refers to self-reported use of any pain medication during menstruation; the indication was not recorded. (—) indicates not applicable or not calculated, as appropriate.

**Table 2:** Ovarian volumes by dysmenorrhea status.

Parameter	Dysmenorrhea (76) Median (IQR)	Control (42) Median (IQR)	*p* Value	r
Right ovarian volume (cm^3^)	8.54 (5.21–13.15)	6.04 (4.23–7.84)	<0.001	0.377
Left ovarian volume (cm^3^)	8.12 (6.02–11.16)	6.07 (4.56–7.51)	<0.001	0.397
Total ovarian volume (cm^3^)	17.04 (12.30–23.48)	11.61 (9.00–15.72)	<0.001	0.437

Data are median (IQR); all three volume parameters were non-normally distributed, so means and standard deviations are not reported. IQR = interquartile range. *p* Values from the Mann–Whitney U test; r is the rank-biserial correlation.

**Table 3:** Spearman rank correlations between ovarian volume parameters and clinical outcomes.

Outcome	ROV ρ (*p*)	LOV ρ (*p*)	TOV ρ (*p*)
VAS score	0.286 (0.002)	0.324 (<0.001)	0.334 (<0.001)
SF-MPQ total score	0.368 (<0.001)	0.330 (<0.001)	0.354 (<0.001)
SF-12 physical summary index (study-specific)	−0.298 (0.001)	−0.292 (0.001)	−0.318 (<0.001)
SF-12 mental summary index (study-specific)	−0.140 (0.132)	−0.157 (0.092)	−0.163 (0.079)
PSQI global score	0.166 (0.101)	0.193 (0.056)	0.203 (0.044)

All coefficients are Spearman’s ρ with two-tailed *p* values. PSQI correlations are based on 99 participants with a complete global score.

**Table 4:** Primary and exploratory nested multivariable logistic regression models for dysmenorrhea, all fitted on the same complete-case sample (*n* = 97).

Variable	Adjusted OR (95% CI)	*p* Value
Model A—Primary adjusted model		
TOV (per 5 cm^3^)	1.764 (1.270–2.681)	0.003
Age (years)	1.325 (1.082–1.654)	0.009
BMI (kg/m^2^)	0.965 (0.845–1.102)	0.585
Age at menarche (years)	0.782 (0.499–1.175)	0.255
Irregular cycle (ref: regular)	1.360 (0.446–4.388)	0.594
Model B—Exploratory: Model A + SF-12 indices		
TOV (per 5 cm^3^)	1.559 (1.153–2.303)	0.010
Age (years)	1.336 (1.050–1.736)	0.022
BMI (kg/m^2^)	0.945 (0.800–1.092)	0.468
Age at menarche (years)	0.766 (0.457–1.240)	0.289
Irregular cycle (ref: regular)	2.394 (0.688–9.202)	0.182
SF-12 physical summary index (study-specific)	0.951 (0.917–0.980)	0.003
SF-12 mental summary index (study-specific)	0.984 (0.955–1.014)	0.298
Model C—Exploratory: Model B + PSQI global score		
TOV (per 5 cm^3^)	1.626 (1.190–2.464)	0.007
Age (years)	1.337 (1.045–1.747)	0.025
BMI (kg/m^2^)	0.967 (0.812–1.125)	0.678
Age at menarche (years)	0.728 (0.428–1.192)	0.218
Irregular cycle (ref: regular)	2.730 (0.766–10.947)	0.134
SF-12 physical summary index (study-specific)	0.948 (0.911–0.978)	0.002
SF-12 mental summary index (study-specific)	0.975 (0.943–1.007)	0.130
PSQI global score	0.827 (0.657–1.026)	0.092

CI = confidence interval; OR = odds ratio. Model A (primary adjusted model): AIC 120.1; McFadden R^2^ 0.162; Nagelkerke R^2^ 0.263; apparent AUC 0.779, optimism 0.047, optimism-corrected AUC 0.733. Model B (exploratory): AIC 102.9; McFadden R^2^ 0.326; Nagelkerke R^2^ 0.478; corrected AUC 0.820. Model C (exploratory): AIC 101.9; McFadden R^2^ 0.349; Nagelkerke R^2^ 0.505; corrected AUC 0.828. Likelihood-ratio tests: A vs. B *p* < 0.001; B vs. C *p* = 0.085; A vs. C *p* < 0.001. Optimism was estimated over 1000 bootstrap resamples. Maximum variance inflation factor: 1.45.

**Table 5:** Exploratory PSQI component and global score comparisons by dysmenorrhea status.

PSQI Component	Dysmenorrhea	Control	*p* Value	r
C1—Subjective sleep quality	1.0 (1.0–2.0)	1.0 (1.0–2.0)	0.693	0.039
C2—Sleep latency	1.0 (1.0–2.0)	1.0 (1.0–2.0)	0.491	0.072
C3—Sleep duration	1.0 (0.0–2.0)	1.0 (0.0–2.0)	0.692	0.043
C4—Habitual sleep efficiency	0.0 (0.0–1.0)	0.0 (0.0–0.0)	0.203	0.114
C5—Sleep disturbances	1.5 (1.0–2.0)	1.0 (1.0–2.0)	0.045	0.196
C6—Use of sleep medication	0.0 (0.0–0.0)	0.0 (0.0–0.0)	0.345	0.040
C7—Daytime dysfunction	2.0 (1.0–2.0)	1.0 (0.0–2.0)	0.001	0.345
Global PSQI score	7.0 (5.0–9.0)	6.0 (5.0–9.0)	0.449	0.091

Data are median (IQR). *p* Values are from the Mann–Whitney U test; r is the rank-biserial correlation. Component 4 and the global score are available for 99 participants; 19 participants lacked usable bed-time and wake-time data. Component-level analyses are exploratory and were not used for primary inference. No adjustment for multiple comparisons was applied.

## Data Availability

The de-identified dataset, the analysis script, and the results file generated by that script are available from the corresponding author on reasonable request. They are not publicly deposited because the study population is small and geographically defined, so open release would carry a residual risk of re-identification. Requests should be addressed to the corresponding author and must state the intended analysis. Each request is assessed jointly by both authors and referred to the Health Sciences Research Ethics Committee of Sivas Cumhuriyet University, which determines whether release is consistent with the consent given by participants; data are then shared under a data-use agreement that prohibits attempts at re-identification and onward transfer. Direct identifiers and the code-to-identity key are never released.

## Supplementary Materials

The following supporting information can be downloaded at https://www.mdpi.com/article/10.3390/diagnostics16213635/s1: Figure S1: PSQI global score by dysmenorrhea status. Figure S2: PSQI component scores by dysmenorrhea status. Table S1: STROBE checklist and selected STARD reporting items for the exploratory ROC analysis.
