# Supplementary material for: Total Ovarian Volume and Multidimensional Pain in Primary Dysmenorrhea: A Cross-Sectional Ultrasound Study
Source: Diagnostics (Basel). 2026 Nov 3;16(21):3635. doi: 10.3390/diagnostics16213635 (PMC13762035; doi:10.3390/diagnostics16213635)
Supplement: Supplementary file 1 [file diagnostics-16-03635-s001.zip › diagnostics-4641031-supplementary.pdf]

Supplementary Materials

Total Ovarian Volume and Multidimensional Pain in Primary Dysmenorrhea: A Cross-Sectional Ultrasound Study

Gamze Sönmez Ünal and Nazan Yurtcu

Figure S1. PSQI global score by dysmenorrhea status

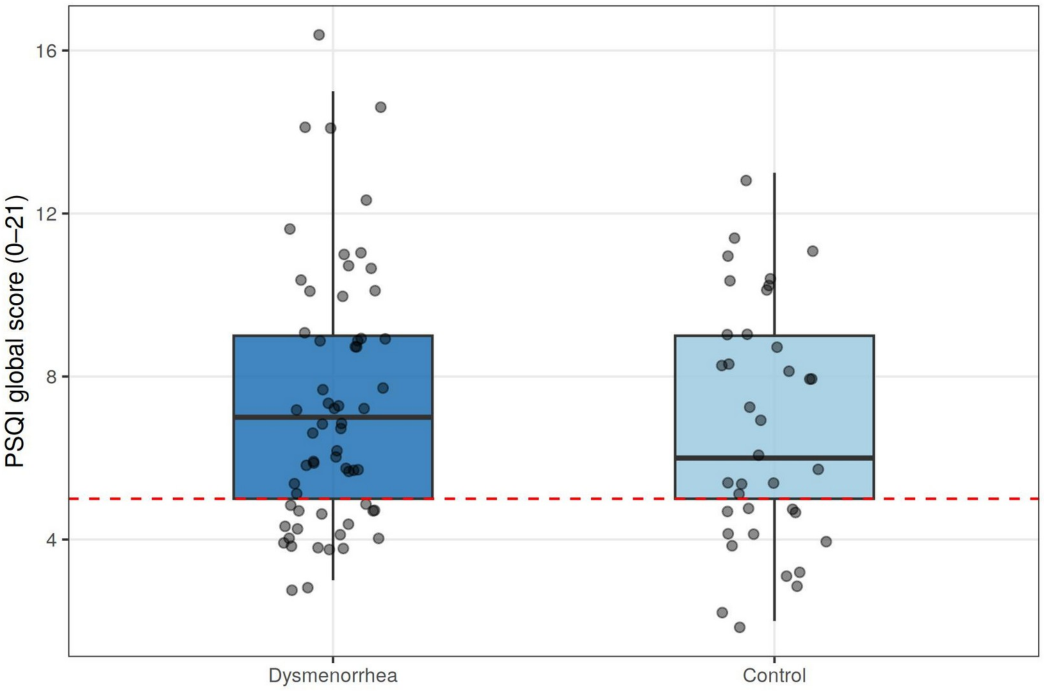

Figure S1. Pittsburgh Sleep Quality Index (PSQI) global score by dysmenorrhea status. Box plots show medians and interquartile ranges with jittered individual observations; the red dashed line marks the conventional threshold of 5, above which sleep quality is regarded as clinically poor. Dysmenorrhea group 7.0 (5.0–9.0), control group 6.0 (5.0–9.0); Mann–Whitney U test $p = 0.449$; rank-biserial $r = 0.091$. Scores are available for 99 participants, since a global score is defined only when all seven components are present.

Figure S2. PSQI component scores by dysmenorrhea status

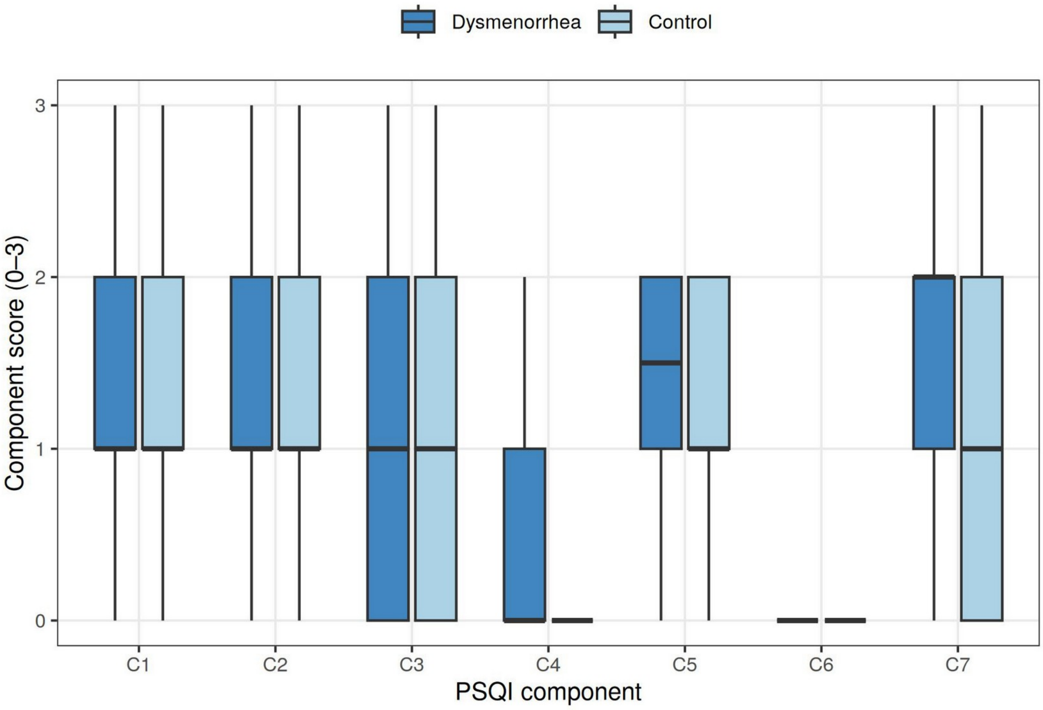

Figure S2. Pittsburgh Sleep Quality Index component scores (C1–C7, each 0–3) by dysmenorrhea status. Component-wise tests are given in Table 5 of the main text. Daytime dysfunction (C7) showed the largest between-group difference ($p = 0.001$; $r = 0.345$), and sleep disturbances (C5) showed a small difference ($p = 0.045$; $r = 0.196$); the remaining components did not differ between groups. Component-level analyses are exploratory, and no adjustment for multiple comparisons was applied.

Table S1. STROBE checklist and selected STARD reporting items for the exploratory ROC analysis.

Item	Recommendation	Reported in
**Part A. STROBE checklist (cross-sectional studies)**		
1a	Indicate the study design with a commonly used term in the title or abstract	Title; Abstract
1b	Provide an informative and balanced summary of what was done and what was found	Abstract
2	Explain the scientific background and rationale	Section 1
3	State specific objectives	Section 1 (final paragraph)
4	Present key elements of study design early in the paper	Section 2.1
5	Describe the setting, locations, and relevant dates, including recruitment and data collection	Sections 2.1 and 2.2
6a	Give the eligibility criteria and the sources and methods of selection of participants	Section 2.2
7	Define all outcomes, exposures, potential confounders, and effect modifiers	Sections 2.2–2.5
8	Give sources of data and details of methods of assessment (measurement) for each variable	Sections 2.3 and 2.4
9	Describe any efforts to address potential sources of bias	Sections 2.2 and 2.4; Strengths and Limitations
10	Explain how the study size was arrived at	Section 2.2 (Sample size)
11	Explain how quantitative variables were handled in the analyses	Sections 2.3 and 2.5
12a	Describe all statistical methods, including those used to control for confounding	Section 2.5
12b	Describe any methods used to examine subgroups and interactions	Not performed; restricted analysis reported as sensitivity analysis (Section 3.4)
12c	Explain how missing data were addressed	Sections 2.3 and 2.5; Figure 1
12d	If applicable, describe analytical methods taking account of sampling strategy	Not applicable (consecutive clinic-based sample; Section 2.2)
12e	Describe any sensitivity analyses	Sections 2.2 and 3.4
13a	Report numbers of individuals at each stage of the study	Section 2.2; Figure 1
13b	Give reasons for non-participation at each stage	Section 2.2; Figure 1
13c	Consider use of a flow diagram	Figure 1
14a	Give characteristics of study participants and information on exposures and potential confounders	Section 3.1; Table 1
14b	Indicate the number of participants with missing data for each variable of interest	Sections 2.3 and 2.5; Table 1 footnote; Figure 1
15	Report numbers of outcome events or summary measures	Sections 3.1–3.2; Tables 1 and 2
16a	Give unadjusted estimates and, if applicable, confounder-adjusted estimates with their precision	Sections 3.2–3.4; Tables 2–4
16b	Report category boundaries when continuous variables were categorized	Section 2.2 (VAS threshold); Section 2.5 (TOV per 5 cm^3)
16c	If relevant, consider translating estimates of relative risk into absolute risk	Not applicable
17	Report other analyses done (subgroups, interactions, sensitivity analyses)	Sections 3.4, 3.5, and 3.8; Table 5
18	Summarise key results with reference to study objectives	Section 4 (first paragraph)
19	Discuss limitations of the study, taking into account sources of potential bias or imprecision	Strengths and Limitations
20	Give a cautious overall interpretation of results	Sections 4 and 5
21	Discuss the generalisability (external validity) of the study results	Section 2.5; Strengths and Limitations
22	Give the source of funding and the role of the funders	Funding statement
**Part B. Selected STARD 2015 items (exploratory ROC analysis of total ovarian volume)**		
10a	Index test, in sufficient detail to allow replication	Section 2.4
10b	Reference standard (definition of the target condition), in sufficient detail to allow replication	Section 2.2
11	Rationale for choosing the reference standard	Section 2.2
12a	Definition of and rationale for test positivity cut-offs, distinguishing pre-specified from exploratory	Section 2.5 (data-derived Youden cut-off; exploratory)
13a	Whether clinical information and reference standard results were available to the performers of the index test	Strengths and Limitations (operator not blinded)
14	Methods for estimating or comparing measures of diagnostic accuracy	Section 2.5
19	Flow of participants, using a diagram	Figure 1
20	Baseline demographic and clinical characteristics of participants	Table 1
24	Estimates of diagnostic accuracy and their precision (such as 95% confidence intervals)	Section 3.5; Figure 5
26	Study limitations, including sources of potential bias, statistical uncertainty, and generalisability	Strengths and Limitations
27	Implications for practice, including the intended use and clinical role of the index test	Section 4

Item wording is abbreviated from the STROBE statement (von Elm et al., Lancet 2007) and the STARD 2015 statement (Bossuyt et al., BMJ 2015), cited as references 18 and 19 in the main text. Section numbers refer to the published article. STARD items not listed were not applicable to this exploratory analysis.

Data and code availability

The de-identified dataset, the analysis scripts used for data preparation, scoring, modelling, and figure generation, and the results file generated by these scripts are available from the corresponding author on reasonable request, as described in the Data Availability Statement of the main text.
